# Efficacy of an Experimental CPP-ACP and Fluoride Toothpaste in Prevention of Enamel Demineralization: An In Vitro Study on Bovine Enamel

**DOI:** 10.1155/ijod/5598592

**Published:** 2025-03-18

**Authors:** Zahra Moslehitabar, Hossein Bagheri, Abdolrasoul Rangrazi, Ali Faramarzi Garmroodi, Aliakbar Hodjatpanah Montazeri

**Affiliations:** ^1^Department of Periodontics, School of Dentistry, Mashhad University of Medical Sciences, Mashhad, Iran; ^2^Dental Materials Research Center, Mashhad University of Medical Sciences, Mashhad, Iran; ^3^Dental Research Center, Mashhad University of Medical Sciences, Mashhad, Iran; ^4^Independent Researcher, Mashhad, Iran

**Keywords:** CPP-ACPF, demineralization, microhardness, toothpaste, white spot lesions

## Abstract

**Objective:** This study evaluated the effects of adding casein phosphopeptide-amorphous calcium phosphate (CPP-ACP), with and without fluoride, to a laboratory toothpaste on the inhibition of enamel demineralization under pH cycling conditions.

**Methods and Materials:** A total of 44 enamel blocks were prepared. Samples were randomly divided into four groups: Group 1—Basic laboratory toothpaste as control; Group 2—Laboratory toothpaste containing 1100 ppm sodium fluoride; Group 3—Laboratory toothpaste containing 1% w/w CPP-ACPF; Group 4—Laboratory toothpaste containing 1% w/w CPP-ACP. Half of each enamel block was coated with nail varnish (sound area), and the other half was subjected to pH cycling for 10 days. During this period, the samples were immersed in a demineralization solution for three separate periods of 1 h each (3 h in total). In the remaining intervals, they were immersed in a remineralization solution (21 h in total). After the first step of demineralization, samples were immersed in an aqueous solution of the toothpaste and distilled water for 6 min. Vickers microhardness was measured at depths of 20, 50, and 120 μm.

**Results:** The relative demineralization (rDEM) index in all treatment groups was significantly smaller than that in the control group. Binary analysis showed that there was no significant difference in the rDEM among the treatment groups, regardless of depth. Tukey's post hoc test revealed that the amount of hardness reduction due to the pH cycle was significant in all groups and at all depths, except for Group 4 at 50 and 120 μm depth. Therefore, CPP-ACP is more effective in preventing demineralization.

**Conclusion:** All three remineralizing agents inhibited enamel demineralization; however, CPP-ACP was more effective at depths of 50 and 120 μm.

## 1. Introduction

Dental caries is a prevalent dental condition characterized by the gradual and persistent deterioration of the hard tissues of the teeth caused by acid produced by bacteria in dental plaque [1]. This acid leads to the breakdown of dental tissues, particularly at the junction between plaque and the tooth surface [2].

The prevention of early carious lesions and stopping their progression are important strategies in minimally invasive dentistry. In a neutral environment, the hydroxyapatite crystals of enamel are in a balanced state with calcium and phosphate ions. Tooth decay occurs when this balance is disrupted, leading to demineralization. Maintaining this balance through a dynamic process at the tooth and biofilm interface is crucial [3].

Prevention is the best approach for caries management. Demineralization can be reversed by neutralizing the oral pH and ensuring that sufficient calcium and phosphate ions are available in the immediate environment [4]. The most crucial preventive measure is to enhance the resistance of enamel against acid attacks. Dental biofilm can absorb calcium, phosphate, and fluoride from saliva and other external sources, which helps in remineralizing enamel after demineralization. External sources of calcium, phosphate, and fluoride can alter the cariogenic potential of dental biofilm [5].

The relationship between fluoride and the prevention of tooth decay has been widely studied. Regular exposure to fluoride is considered the most effective intervention for limiting tooth decay [6]. The scientific basis for using fluoride against dental caries is that fluoride ions can enter the crystalline structure of dental hard tissues, reducing their solubility and making them more resistant to acids. Fluoride ions replace the hydroxyl groups in hydroxyapatite, forming fluorapatite [7]. While fluoride inhibits further demineralization of enamel, it also hinders the uptake of calcium and phosphate ions, which are necessary for repairing deep lesions [8].

In recent years, studies have recommended the use of other preventive products. One of these is pastes containing casein phosphopeptide-amorphous calcium phosphate (CPP-ACP), which are marketed under the commercial names MI Paste and MI Paste Plus. This complex was first introduced by Reynolds et al. [9] at the Melbourne School of Dentistry.

CPP is a milk-derived protein containing the cluster sequence—Ser (P)-Ser (P)-Ser (P)-Glu-Glu-, which can bind calcium and phosphate ions and stabilize them as ACP. In the oral environment, CPP-ACP adheres to plaque, hydroxyapatite, and soft tissues. It delivers bioavailable calcium and phosphate into saliva and dental plaque, aiding in remineralization. Additionally, studies have shown that CPP is a suitable carrier for fluoride ions and can increase the depth of fluoride penetration in demineralized enamel. CPP-ACP and CPP-ACPF may also function as buffer systems, reducing the destructive impact of acids [10]. In vitro studies demonstrate that when placed on the enamel surface, CPP-ACP interacts with hydrogen ions and can diffuse into the enamel, producing subsurface mineral gains [11].

In the past few years, numerous studies have examined the efficacy of CPP-ACP in preventing and treating superficial dental caries and erosion, as well as addressing white spot lesions (WSLs). In some laboratory and animal studies, the remineralization potential of CPP-ACP has been established. ACP has been integrated into chewing gums and gels to deliver calcium to dental biofilms and the enamel surface [12]. However, a recent systemic review suggests that CPP-ACP treatment is only effective in the early stages of WSLs due to its limited ability to penetrate [13, 14].

If CPP-ACP proves to have similar protective properties as fluoride toothpastes, it could be used in various preventive dentistry and daily oral hygiene applications. Therefore, the goal of this laboratory study was to assess the protective effects of CPP-ACP/CPP-ACPF-containing toothpastes on enamel demineralization and comparing them to fluoride toothpastes.

The hardness of dental enamel is closely linked to its chemical composition [15]. Studies have shown that regions with higher concentrations of calcium and phosphorus exhibit greater microhardness values, while areas with lower levels of these minerals tend to have correspondingly lower microhardness values. Transverse microradiography (TMR) is considered the gold standard for assessing mineral content, lesion depth, and surface layer characteristics in enamel lesions. Conversely, cross-sectional hardness evaluates the mechanical strength of enamel, which TMR cannot measure [16]. Previous research has demonstrated a correlation between mineral content determined through TMR and enamel hardness assessed by cross-sectional hardness [17–19]. The surface hardness (SH) test, which has been utilized in some research, has proven capable of detecting the initial phases of enamel demineralization on the surface of the enamel. However, it is not capable of measuring the depth of the lesion. Since previous studies have investigated the effects of CPP-ACP and fluoride on the surface of the enamel, the aim of this study is to measure the effect of these compounds in the deeper areas of the enamel. Therefore, we opted to employ the cross-section hardness (CSH) method to assess the depth of the lesion. This technique is widely recognized for evaluating enamel and can be applied to enamel block lesions [20]. The null hypothesis is that CPP-ACP and fluoride have no effect on the prevention of enamel demineralization.

## 2. Materials and Methods

### 2.1. Sample Preparation

In this study, enamel specimens were prepared from freshly extracted bovine permanent mandibular incisors. Before starting the experiment, the sample size was calculated according to a prior study [16]. This study, taking into account a power level of 80% and a significance level of 0.05, with a type I error (*α*) of 0.05 and a type II error (*β*) of 0.2, determined that at least 10 specimens per subgroup were needed. Therefore, 11 specimens were chosen for each group in the current study.

The teeth were cleaned of debris and adherent soft tissues using a scaler (LM-Dental, Parainen, Finland) and stored in a 0.1% thymol solution (pH 7.0) for 3 days. They were kept in normal saline until the initiation of the experiment. The teeth were evaluated using a stereomicroscope (Dino Lite Pro, Anmo Electronic, New Taipei City, Taiwan) at ×10 magnification to detect cracks, caries, fluorosis, calcification, discoloration, hypomineralization, and other anomalies. Specimens were prepared by separating the roots and sectioning the crown of selected teeth into 10 mm × 10 mm (width × length) enamel blocks using a low-speed diamond-coated band saw (Isomet, Buehler, Lake Bluff, IL, USA) with water as a coolant.

**Figure 1:**
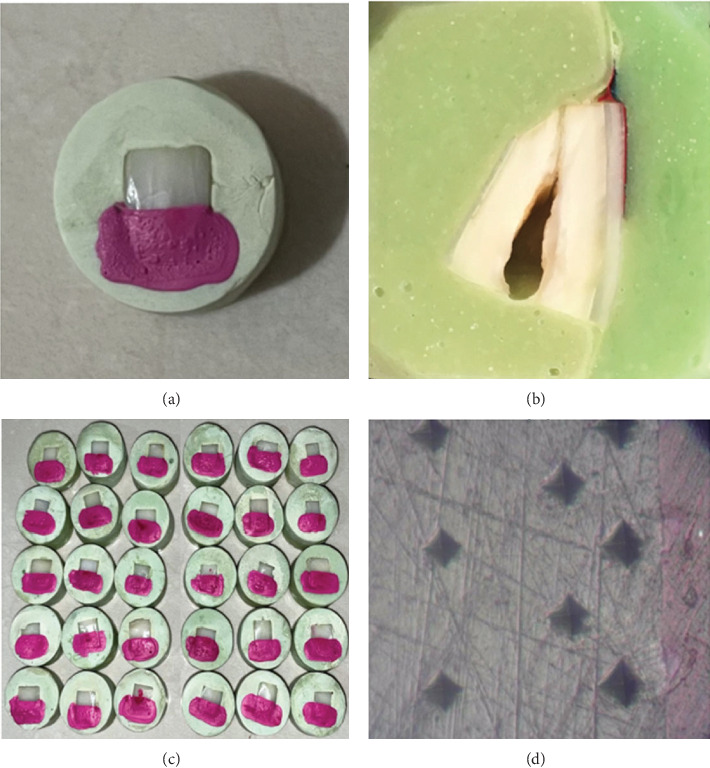
(a and c) Samples before the treatment process, (b) samples prepared for microhardness measurements, and (d) Vicker's microhardness measurements at depths of 20, 50, and 120 μm.

A total of 44 enamel blocks were obtained from healthy teeth and mounted in self-cure acrylic resin (Acropars, Kaveh, Tehran, Iran) with all block surfaces embedded except the enamel surface. The enamel surfaces were sequentially polished with 600, 800, 1200, 2500, and 5000 grit sandpaper (Starcke, Melle, Germany) under running water to attain flat, mirror-like surfaces. Then, the surface of each specimen was divided into two equal areas, and one area was covered with acid-resistant nail varnish to create a protected enamel area (negative control).  Figure 1a shows a sample before the treatment process.

### 2.2. pH Cycling and Treatment With Dentifrices

Before starting the pH cycles, each enamel sample was immersed in 10 ml of artificial saliva for 24 h (pH 6.5). The enamel specimens then underwent pH cycling for 10 days at 37°C. Each day, the specimens were immersed individually in 10 ml of demineralizing solution for 6–7 h, 14–15 h, and 22–23 h, and were placed in the remineralizing solution for the remaining 21 h. The demineralization solution contained an acid buffer with 2.2 mM CaCl_2_ (Merck, Darmstadt, Germany), 2.2 mM Na_2_HPO_4_ (Merck, Darmstadt, Germany), and 50 mM acetate at pH 4.5. The remineralizing solution contained 3 mM CaCl_2_ (Merck, Darmstadt, Germany), 5 mM Na_2_HPO_4_ (Merck, Darmstadt, Germany), and 50 mM NaCl (Merck, Darmstadt, Germany) at pH 6.5. To prepare the solutions in distilled water, the chemicals were measured using a precision scale (AND, Tokyo, Japan) with a 0.0001 g accuracy and mixed with a magnetic stirrer.

The specimens were exposed to the chosen dentifrice slurries (a 1:3 toothpaste to deionized water ratio, w/w) once a day for 6 min after the first demineralization stage. A standardized volume was used for each group, and all specimens were immersed in fresh solution. After the last demineralization, the specimens were immersed in remineralizing solution for another 14 h. The solutions were renewed every 48 h. At every transfer between the different solutions, all specimens were rinsed in distilled water for 1 min before and after any solution change or dentifrice slurry application, and they were wiped dry with a soft paper towel.

A basic laboratory toothpaste was made at the Dental Materials Research Center of Mashhad University of Medical Sciences. The basic toothpaste used in this study contains 40% calcium carbonate (Merck, Darmstadt, Germany), 3% carboxymethyl cellulose (Sigma–Aldrich, St. Louis, USA), 30% Glycerol (Merck, Darmstadt, Germany), 0.2% methylparaben (Merck, Darmstadt, Germany), 1.5% sodium lauryl sulfate (Merck, Darmstadt, Germany), and 25% distilled water. After preparing the basic toothpaste, three types of specific toothpaste were formulated by adding active agents: 1% w/w CPP-ACP, 1% w/w CPP-ACPF, and 1100 ppm NaF (Merck, Darmstadt, Germany).

The 44 enamel samples were randomly divided into four groups:

  Group 1: Basic laboratory toothpaste (control).
  Group 2: Laboratory toothpaste containing 1100 ppm sodium fluoride.
  Group 3: Laboratory toothpaste containing 1% w/w CPP-ACPF.
  Group 4: Laboratory toothpaste containing 1% w/w CPP-ACP.

CPP-ACP was synthesized according to previous studies [21].

### 2.3. Microhardness Measurements

To perform a cross-sectional microhardness test, each sample was embedded in acrylic resin (Acropars, Kaveh, Tehran, Iran), ensuring that the enamel surface was entirely covered by resin. Subsequently, the samples were sectioned perpendicularly to the surface through the center so the cross-sectional surfaces of both the intervention and sound areas were accessible and polished as described previously (Figure 1b).

The Vickers microhardness testing method was applied using a 100-g load for 10 s. Indentations were made at 20, 50, and 120 μm from the outer enamel surface using a Vickers microhardness tester (Koopa Pajoohesh, Sari, Iran). Three points were measured at each distance, and the mean values were calculated (Figure 1c,d). Thus, the microhardness profile was obtained for both areas. The relative demineralization (rDEM) index was measured using the following formula:

  $$\begin{array}{c} \text{rDEM}=\frac{\text{VHN}\left(x\right)-\text{VHN}\left(s\right)}{\text{VHN}\left(s\right)}\times 100. \end{array}$$

### 2.4. Statistical Analysis

To evaluate the presence of significant differences among the groups, data were analyzed using a two-way analysis of variance (ANOVA), followed by Tukey's post hoc test. Differences between baseline and post-pH cycling mean enamel surface microhardness values were analyzed using paired Tukey tests. Statistical analysis was performed using SPSS software version 22 (SPSS Inc., IBM Corp., Armonk, New York, USA). Statistical significance was set at *p* < 0.05.

### 2.5. Ethical Approval

This study was approved by the Ethics Committee of Mashhad University of Medical Sciences, Mashhad, Iran (Ethical code: IR.MUMS.DENTISTRY.REC.1401.071).

## 3. Results

**Figure 2:**
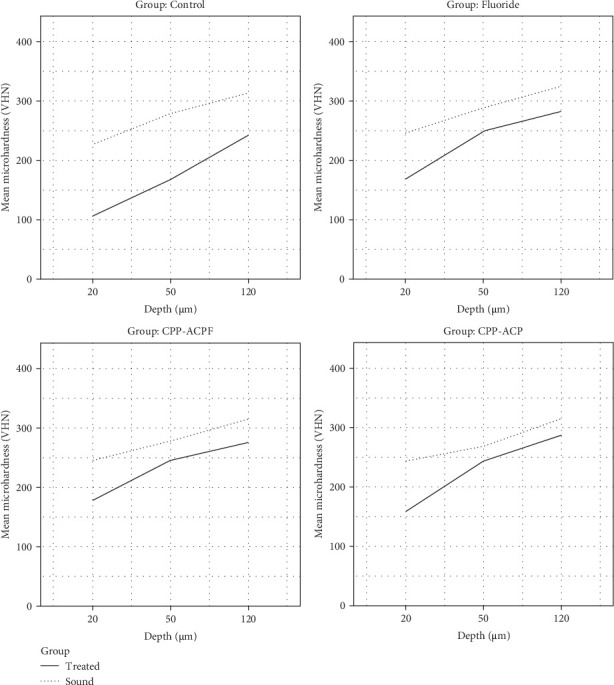
Mean microhardness values at different depths.

Table 1 and Figure 2 present the mean enamel microhardness values at three distinct depths for both the sound and intervention areas across the four groups. In each figure, the dotted lines indicate the sound area, while the dashed lines denote the intervention area. A smaller gap between these lines signifies a greater impact of the toothpaste used in that group. It is evident that all three treatment groups exhibit greater effectiveness compared to the control group, and the pattern of microhardness changes in the treatment groups differs from that of the control group.

As shown in Table 2, repeated measures ANOVA was performed to determine the effect of pH cycles on enamel demineralization for each group separately. The results showed that in all groups, microhardness decreased due to the demineralization cycle. Additionally, in all treatment groups, depth had a significant effect on demineralization behavior (*p* < 0.05), whereas in the control group, depth did not have a significant effect on demineralization behavior (*p* = 0.064). This indicates that demineralization occurred at all depths, but the degree of demineralization varied at different depths in the treatment groups.

As presented in Table 3, Tukey's post hoc test revealed that the microhardness level decreased due to the demineralization cycle across all groups and depths. However, in the CPP-ACP group, the reduction in microhardness was not significant at depths of 50 μm (*p* = 0.086) and 120 μm (*p* = 0.061). This indicates that CPP-ACP is more effective in preventing demineralization at these depths compared to the other groups.

For each depth, the relative demineralization index (rDEM = ((VHN (*x*) – VHN(*s*))/VHN(*s*)) × 100) was calculated. The rDEM data are summarized in Table 4.

**Figure 3:**
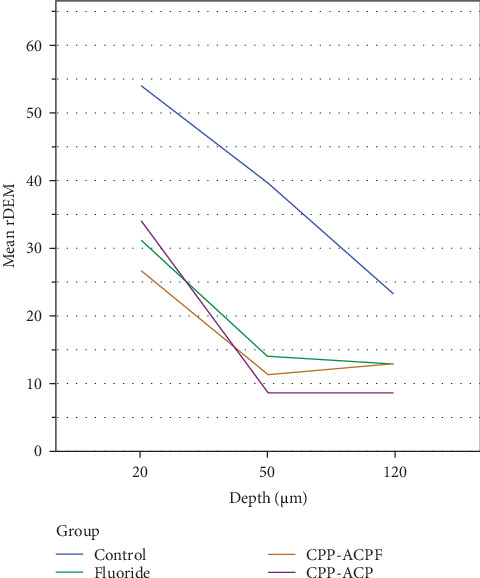
Mean relative demineralization index according to depth.

As shown in Figure 3, the demineralization level in the control group was higher than in the treatment groups. In the control group, demineralization decreases linearly from the surface to a depth of 120 μm, whereas in the treatment groups, demineralization occurs up to a depth of 50 μm and then remains constant.

Based on Table 5, two-way ANOVA showed that both the treatment group (the effective factor in each group) and depth had a significant effect on the rDEM index (*p* < 0.001). However, the interaction effect between group and depth was not significant (*p* = 0.24).

Two-way ANOVA showed that both the treatment group (the effective factor in each group) and depth had a significant effect on the rDEM index (*p* < 0.001). However, the interaction effect between group and depth was not significant (*p* = 0.24).

Therefore, a pairwise analysis of the groups was conducted based on depth and treatment group (Table 6). The results showed that, regardless of depth, the level of demineralization in the treatment groups was significantly lower compared to the control group, with no statistically significant difference between the treatment groups.

As shown in Table 7, the pairwise comparison of the data based on depth showed that, regardless of the type of treatment group, the level of mineralization at a depth of 20 μm was significantly higher. However, there was no significant difference between the depths of 50 and 120 μm.

## 4. Discussion

CPP-ACP serves as a reservoir for calcium and phosphate. By binding to the calcium and phosphate in enamel, CPP stabilizes ACP. When the pH of plaque drops, CPP releases calcium and phosphate ions, creating supersaturation. This process reduces demineralization and promotes remineralization [22]. The role of fluoride in increasing tooth resistance against acid attacks has been well established. Fluoride has also been added to CPP-ACP to enhance its properties. Previous research demonstrated that CPP not only enhances the absorption of fluoride into plaque but also promotes the absorption of fluoride into deeper layers of enamel [9]. CPP-ACP has been used in various dental care products and therapeutic materials, such as mouth rinses, sugar-free chewing gums, and sports drinks, to decrease enamel demineralization [23]. With this in mind, the study aimed to assess the protective effectiveness of toothpaste containing CPP-ACP with and without fluoride, as well as toothpaste containing NaF, in preventing enamel demineralization.

Although human teeth are generally used for in vitro research, this study used bovine teeth because they are readily available in large quantities and are in good condition. Bovine teeth also have a more consistent composition compared to human teeth and provide larger untreated flat surfaces for testing. Additionally, the distribution of minerals in carious lesions in bovine teeth is reported to be similar to that in human teeth, and the structural changes observed in both are comparable [24].

This research utilized pH cycling to mimic the conditions of the oral environment. In various studies, the time samples spent in the demineralizing agent and artificial saliva differed, spanning from 5 to 28 consecutive days, with immersion in the demineralizing agent lasting between 3 and 6 h each day [20, 25]. In this study, the samples were immersed for 21 h per day in artificial saliva with a pH of 6.5 and for 3 h in a demineralizing agent with a pH of 4.5 over a period of 10 days. Previous studies have reported varying pH values for the demineralizing agent, ranging from 3.5 to 5 [26].

The results of this study demonstrated that all three mineralizing agents can significantly prevent enamel demineralization at all examined depths, and therefore, the null hypothesis was rejected. At a depth of 20 μm, all three groups exhibited less demineralization compared to the control group, but there was no significant difference among the three groups. However, CPP-ACP showed a greater protective effect at depths of 50 and 120 μm. This finding aligns with previous studies. Pithon et al. [27] reported that, compared to Duraphat varnish, MI varnish is more effective in reducing the depth of caries lesions. In a randomized double-blind in situ study, Robertson et al. [28] demonstrated that MI Paste Plus not only prevents the formation of new WSLs during orthodontic treatment but also reduces the number of existing WSLs. Unlike our findings, Rangarajan et al. [22] assessed the clinical effectiveness of MI varnish (CPP-ACP) and Fluoritop varnish (5% NaF) in 30 orthodontic patients and discovered no significant difference in preventing WSLs between the two varnishes, except in the cervical area where MI varnish proved to be more effective than Fluoritop. However, in a clinical and laboratory study conducted by Uysal et al. [29], the effects of fluoride and CPP-ACP gel on the prevention of demineralization around orthodontic brackets were investigated in the occlusal and cervical areas at a depth of 10 μm from the outer enamel surface. After measuring hardness, the study concluded that there was no significant difference between the cervical and occlusal areas. It was also noted that both fluoride and CPP-ACP effectively prevented demineralization compared to the control group, though no statistically significant difference was observed. This inconsistency in the results of the studies can be due to the difference in the method of conducting the study and data measurement. In a systematic review conducted by Yazarloo et al. [30], various methods for the prevention and treatment of WSLs were evaluated. Their study established that maintaining oral hygiene is crucial for preventing enamel lesions, and using fluoride-containing toothpaste twice a day yields results comparable to the use of MI Paste.

Tahmasebi et al. [5] compared sodium fluoride, MI Paste Plus, and Remin Pro toothpaste in a study on the prevention of WSLs. They found that sodium fluoride is more effective than Remin Pro and MI Paste Plus in preventing tooth demineralization. This conclusion was based on measurements of enamel SH. The aim of the current study was to establish a remineralization regimen that is effective in both the surface and deep layers of enamel. Fluoride primarily affects the surface layer, forming a relatively impermeable remineralized layer, which limits remineralization in the subsurface region. Consequently, fluoride alone cannot prevent the progression of demineralization within the lesion body, leading to continued subsurface enamel destruction. In contrast, CPP-ACP exerts its protective effect at greater depths by penetrating the lesion and inhibiting hydroxyapatite dissolution. This is why the remineralization pattern in the CPP-ACP group differs from those in the fluoride and CPP-ACPF groups. ACP penetrates the lesion body, concentrating calcium there and reducing hydroxyapatite dissolution. However, with CPP-ACPF, the existing fluoride quickly forms fluorapatite on the surface, blocking the superficial layer and inhibiting CPP-ACP penetration into the deeper parts of the lesion. This effect is determined by measuring microhardness at depths of 50 and 120 μm. Additionally, Rafiei et al. [31] investigated the effect of adding CPP-ACP to a daily-use toothpaste on the remineralization of enamel caries lesions. Their results showed a significant difference in the remineralization efficacy between the groups in superficial lesions at a depth of 20 µm. The toothpaste containing both fluoride and CPP-ACP had significantly greater microhardness compared to the other experimental groups (fluoride-containing toothpaste and CPP-ACP-containing toothpaste) [31]. It should be clarified that only superficial lesions will be remineralizable. Remineralization effects will occur only at a molecular level, with no formation of new hydroxyapatite in advanced lesions. This distinction is particularly relevant and important, as many dentists often wonder why advanced lesions do not disappear.

Our in vitro study had some limitations. One was that demineralization was induced using chemical solutions rather than the acidic byproducts of *Streptococcus mutans* bacteria. Despite attempts to replicate an environment similar to the oral cavity, there are notable differences, including the inability to reproduce continuous saliva flow, the ongoing dilution of ions, and the rinsing effect of saliva. Additionally, the antimicrobial effects of these products were not evaluated in this study. Currently, there is a growing number of dental products incorporating CPP-ACP, with potential applications that appear limitless. It is anticipated that CPP-ACP will play a significant role in future dental efforts to combat white spots and decalcification [28]. Further clinical and preclinical studies are needed to evaluate the long-term protective potential of CPP-ACP. Repeating this study with higher concentrations of these products would also be useful.

## 5. Conclusion

All three remineralizing agents inhibited enamel demineralization; however, CPP-ACP was more effective at depths of 50 and 120 μm.

## Figures and Tables

**Table 1:** Mean and standard deviation of microhardness (VHN).

Group	Depth (μm)	Sound		Treated (DEM)	
		Mean	Standard deviation	Mean	Standard deviation
Control	20	229	27	106	42
	50	279	22	167	37
	120	313	17	242	78

Fluoride	20	246	33	168	31
	50	288	25	248	38
	120	325	20	283	45

CPP-ACPF	20	247	31	179	34
	50	279	25	247	40
	120	316	18	276	34

CPP-ACP	20	244	25	158	40
	50	270	23	245	40
	120	315	28	287	44

Abbreviation: CPP-ACP, casein phosphopeptide-amorphous calcium phosphate.

**Table 2:** Results of the repeated measures ANOVA.

Group	Source	*p*
Control	Hardness	<0.0001
	Hardness *⁣*^*∗*^ depth	0.064

Fluoride	Hardness	<0.0001
	Hardness *⁣*^*∗*^ depth	0.026

CPP-ACPF	Hardness	<0.0001
	Hardness *⁣*^*∗*^ depth	0.047

CPP-ACP	Hardness	<0.0001
	Hardness *⁣*^*∗*^ depth	0.006

*Note*: The asterisk (*⁣*^*∗*^) represents the interaction effect, which refers to the simultaneous influence of two variables. Hardness *⁣*^*∗*^ depth indicates the combined effect of “hardness” and “depth”.

Abbreviations: ANOVA, analysis of variance; CPP-ACP, casein phosphopeptide-amorphous calcium phosphate.

**Table 3:** Tukey's post hoc test for sound and exposed areas.

Group	Depth (μm)	(*I*) Hardness	(*J*) Hardness	Mean difference (*I*–*J*)	*p*
Control	20	Sound	Exposure	122.200*⁣*^*∗*^	<0.0001
	50	Sound	Exposure	112.064*⁣*^*∗*^	<0.0001
	120	Sound	Exposure	70.727*⁣*^*∗*^	<0.0001

Fluoride	20	Sound	Exposure	78.145*⁣*^*∗*^	<0.0001
	50	Sound	Exposure	40.436*⁣*^*∗*^	0.001
	120	Sound	Exposure	41.673*⁣*^*∗*^	<0.0001

CPP-ACPF	20	Sound	Exposure	68.333*⁣*^*∗*^	<0.0001
	50	Sound	Exposure	31.800*⁣*^*∗*^	0.005
	120	Sound	Exposure	39.800*⁣*^*∗*^	0.001

CPP-ACP	20	Sound	Exposure	85.920*⁣*^*∗*^	0.000
	50	Sound	Exposure	24.900	0.086
	120	Sound	Exposure	27.320	0.061

Abbreviation: CPP-ACP, casein phosphopeptide-amorphous calcium phosphate.

*⁣* ^*∗*^Statistically significant.

**Table 4:** Mean and standard deviation of the relative demineralization index.

Group	Depth (μm)	Mean	Std. deviation
Control	20	54.1193	15.64325
	50	39.8193	14.60344
	120	22.8967	23.52684

Fluoride	20	31.2138	12.19385
	50	14.0780	11.12222
	120	12.9226	11.88203

CPP-ACPF	20	26.7254	14.68135
	50	11.3047	12.47308
	120	12.7477	8.15714

CPP-ACP	20	34.3062	19.09545
	50	8.7032	16.59872
	120	8.7103	10.93341

Total	20	36.4189	18.36534
	50	18.5354	18.27207
	120	14.4111	15.19590

Abbreviation: CPP-ACP, casein phosphopeptide-amorphous calcium phosphate.

**Table 5:** Two-way analysis of variance on the effect of treatment group and depth on the relative demineralization index.

Tests of between-subjects effects							
Source	Type III sum of squares	df	Mean square	*F*	Sig.	Noncent. parameter	Observed power^b^
Corrected model	24,930.754^a^	11	2266.432	10.500	0.000	115.500	1.000
Intercept	70,320.074	1	70,320.074	325.781	0.000	325.781	1.000
Group	11,138.260	3	3712.753	17.201	0.000	51.602	1.000
Depth	12,290.273	2	6145.137	28.469	0.000	56.939	1.000
Group *⁣*^*∗*^ depth	1748.640	6	291.440	1.350	0.240	8.101	0.512
Error	25,902.088	120	215.851	—	—	—	—
Total	12,1402.350	132	—	—	—	—	—
Corrected total	50,832.842	131	—	—	—	—	—

*Note*: The asterisk (*⁣*^*∗*^) represents the interaction effect, which refers to the simultaneous influence of two variables. Group *⁣*^*∗*^ depth indicates the combined effect of “group” and “depth”.

^a^ *R*-squared = 0.490 (adjusted *R*-squared = 0.444).

^b^Computed using alpha = 0.05.

**Table 6:** The results of Tukeyʼs post hoc analysis for the pairwise comparison of groups in the relative demineralization index based on the treatment group.

(*I*) Group	(*J*) Group	Mean difference (*I* – *J*)	*p*
Control	Fluoride	19.5403*⁣*^*∗*^	<0.0001
	CPP-ACPF	22.0192*⁣*^*∗*^	<0.0001
	CPP-ACP	21.7052*⁣*^*∗*^	<0.0001

Fluoride	Control	−19.5403*⁣*^*∗*^	<0.0001
	CPP-ACPF	2.4789	0.897
	CPP-ACP	2.1649	0.937

CPP-ACPF	Control	−22.0192*⁣*^*∗*^	<0.0001
	Fluoride	−2.4789	0.897
	CPP-ACP	−0.3140	1.000

CPP-ACP	Control	−21.7052*⁣*^*∗*^	<0.0001
	Fluoride	−2.1649	0.937
	CPP-ACPF	0.3140	1.000

Abbreviation: CPP-ACP, casein phosphopeptide-amorphous calcium phosphate.

*⁣* ^*∗*^Statistically significant.

**Table 7:** The results of Tukeyʼs post hoc analysis for the pairwise comparison of groups in the relative demineralization index based on the depth.

(*I*) Depth	(*J*) Depth	Mean difference (*I* – *J*)	*p*
20	50	17.8834*⁣*^*∗*^	<0.0001
	120	22.0078*⁣*^*∗*^	<0.0001

50	20	−17.8834*⁣*^*∗*^	<0.0001
	120	4.1244	0.389

120	20	−22.0078*⁣*^*∗*^	<0.0001
	50	−4.1244	0.389

*⁣* ^*∗*^Statistically significant.

## Data Availability

Data are contained within the article.
